# Maintenance of Mouse Nephron Progenitor Cells in Aggregates with Gamma-Secretase Inhibitor

**DOI:** 10.1371/journal.pone.0129242

**Published:** 2015-06-15

**Authors:** Shunsuke Yuri, Masaki Nishikawa, Naomi Yanagawa, Oak D. Jo, Norimoto Yanagawa

**Affiliations:** 1 Medical and Research Services, Greater Los Angeles Veterans Affairs Healthcare System at Sepulveda, North Hills, California, United States of America; 2 University of California at Los Angeles, David Geffen School of Medicine, Los Angeles, California, United States of America; University of Minnesota Medical School, UNITED STATES

## Abstract

Knowledge on how to maintain and expand nephron progenitor cells (NPC) *in vitro* is important to provide a potentially valuable source for kidney replacement therapies. In our present study, we examined the possibility of optimizing NPC maintenance in the "re-aggregate" system. We found that Six2-expressing (Six2^+^)-NPC could be maintained in aggregates reconstituted with dispersed cells from E12.5 mouse embryonic kidneys for at least up to 21 days in culture. The maintenance of Six2^+^-NPC required the presence of ureteric bud cells. The number of Six2^+^-NPC increased by more than 20-fold at day 21, but plateaued after day 14. In an attempt to further sustain NPC proliferation by passage subculture, we found that the new (P1) aggregates reconstituted from the original (P0) aggregates failed to maintain NPC. However, based on the similarity between P1 aggregates and aggregates derived from E15.5 embryonic kidneys, we suspected that the differentiated NPC in P1 aggregates may interfere with NPC maintenance. In support of this notion, we found that preventing NPC differentiation by DAPT, a γ-secretase inhibitor that inhibits Notch signaling pathway, was effective to maintain and expand Six2^+^-NPC in P1 aggregates by up to 65-fold. The Six2^+^-NPC in P1 aggregates retained their potential to epithelialize upon exposure to Wnt signal. In conclusion, we demonstrated in our present study that the "re-aggregation" system can be useful for *in vitro* maintenance of NPC when combined with γ-secretase inhibitor.

## Introduction

The development of mammalian kidney mainly involves three types of cells, i.e., metanephric mesenchyme (MM), ureteric bud (UB) and stromal mesenchyme (SM) [1]. UB is first induced by MM to extend from the nephric duct and undergoes elaborate branching morphogenesis to give rise to the collecting duct system [2]. In turn, MM is induced by UB to form the remaining nephron structures [3,4]. The SM cells also play important roles in UB branching morphogenesis [5], MM differentiation [6,7] and the formation of vasculature in the kidney [8,9].

MM contains multi-potent self-renewing nephron progenitor cells (NPC), which condenses around the UB tips to form the so called cap mesenchyme [10–12]. The NPC express unique combinations of transcription factors, such as Hox11 paralogs, Osr1, Pax2, Eya1, WT1, Sall1, and Six2, where Six2 and Sall1 were shown to be essential for their progenitor status [10–14]. Upon induction from UB, NPC undergoes mesenchymal-to-epithelial transformation (MET) while migrating from UB tips to sequentially form pre-tubular renal aggregates, renal vesicles, comma- and S-shaped bodies, which further elongate to form the different segments of the nephron. It is generally considered that UB induces NPC differentiation through the secretion of Wnt9b, which activates Wnt4 and Fgf8 in MM cells [15]. SM cells have also been found to cooperate with Wnt9b and promote NPC differentiation through Fat4 which modulates β-catenin signal via the Hippo-YAP pathway [7].

The balance between self-renewal and differentiation of NPC is important in determining the final nephron number, which can have significant health consequences [10–12]. However, our understanding on the mechanism that maintains the proliferation and the progenitor status of NPC remains limited. Available evidence indicates that receptors, such as Fgfr1 and 2, and ligands, such as Fgf9 and 20 [16, 17] and BMP7 [18], all contribute to the maintenance of NPC. Paradoxically, the Wnt9b secreted from UB cells has also been found to contribute to the maintenance of NPC [19]. The UB tips, together with the SM in the periphery, form the *in vivo* niche where NPC are maintained [20]. Further revelations on the mechanisms whereby the progenitor status of NPC is maintained will help not only to advance our knowledge of kidney development and how nephron numbers are determined, but may also enable the design of an *in vitro* niche to allow the expansion of NPC and provide an autologous source for kidney replacement therapies.

Our current studies are aimed at testing the possibility of using the “re-aggregate” system to maintain and expand NPC over a prolonged period of time in *in vitro* culture. The re-aggregation of dispersed embryonic kidney cells has been widely used as a valuable tool for studies on kidney development and organogenesis [21–25]. Dissociating embryonic kidney cells into single cell suspensions and re-aggregating them by centrifugation and culture *in vitro* led to the formation of preliminary nephron structures with UB branching and MM differentiation [21–25]. Our rationale is that the recapitulation of the *in vivo* UB tip niche in the re-aggregates may allow for the maintenance of NPC cultured *in vitro*, as well as for the exploration of the underlying mechanism. We show that NPC could be maintained and expanded for up to 21 days in our re-aggregate system. We found that the developmental stage of MM cells affects the maintenance of NPC in aggregates. When MM cell differentiation was prevented by inhibiting Notch signaling pathway with γ-secretase inhibitor, *N*-*S*-phenyl-glycine-*t*-butyl ester (DAPT), we were able to perform passage subculture of the aggregates and expand NPC up to 65-fold.

## Materials and Methods

### Mice

All mice were obtained from Jackson Laboratory (Bar Harbor, Main). Four lines of mouse, *Six2* ^*TGC/+*^ [4], Six2^*GCE/+*^ [4], *Hoxb7* ^*myr-Venus/+*^ [26] and *Foxd1* ^*GCE/+*^ [27], were maintained with C57BL/6J background and used in our present studies. Mice and embryos were genotyped using the universal PCR genotyping protocols with primers as following: (forward)ACGACGGCAACTACAAGACC/(reverse)TGTAGTTGTACTCCAGCTTGTGC. The morning of the discovery of a vaginal plug was considered as E0.5.

### Dissection, dissociation and re-aggregation of embryonic kidney cells

Mouse embryonic kidneys were dissected from embryos at indicated developmental stages, and cells were dissociated and re-aggregated as previously described [21]. In brief, embryonic kidneys were dissected free-hand using fine needles under a dissecting microscope (Olympus, Center Valley, PA) in Dulbecco's Modified Eagle Medium (DMEM) with 10% fetal bovine serum (FBS) (Invitrogen, Grand Island, NY), and incubated in collagenase (Sigma, St. Louis, MO) at 37°C for 10 min. When indicated, the mesenchyme region was surgically separated from UB with fine needles. To make dispersed cells, the embryonic kidney rudiments were cut into small pieces with scissors and placed in 0.25% Trypsin/EDTA (Invitrogen) in phosphate-buffered saline (PBS; Sigma) at 37°C for 5 min. After adding ice-cold DMEM with 10% FBS, cells were dissociated by trituration and filtered through 70μm cell strainer (BD Bioscience, San Jose, CA). Gentle pipetting was repeated to ensure the suspension of single cells. To re-aggregate the dispersed single cells, a total of 2 x 10^4^ cells were placed into 96 well ultra-low attachment round bottom dish (Corning, Tewksbury, MA), centrifuged at 650*g* for 2 min and cultured overnight.

### Cell culture

The re-aggregated pellets were carefully collected and placed on top of a 0.4 μm isopore polycarbonate filter (Millipore, Billerica, MA), and cultured at 37°C and 5% CO_2_ in keratinocyte serum-free medium (KSFM; Invitrogen) containing 10% FBS (Invitrogen), 10 μM Y27632, a ROCK inhibitor, (Abcam Biochemicals, Cambridge, MA) and 110 μM 2-mercaptoethanol (ME; Invitrogen). Chemicals and growth factors, such as dimethyl sulfoxide (DMSO; Sigma), 1 μg/ml Heparin (Sigma), 200 ng/ml Fgf9 (ProSpec, East Brunswick, NJ) and 50 ng/ml Bmp7 (PeproTech, Rocky Hill, NJ), as well as various inhibitors, such as Notch inhibitor, 5μM DAPT (Cellagen, San Diego, CA), GSK3β inhibitor, 500 nM BIO (Stemgent, Cambridge, MA), Wnt inhibitor, 500 nM IWP2 (Stemgent), JNK inhibitor, 10 μM SP600125 (Tocris Bioscience, Minneapolis, MN) and Bmp inhibitor, 2.5 μM Dorsomorphin (Sigma), were added as indicated.

### Immunocytochemistry and confocal microscope

The aggregates were fixed with 4% Paraformaldehyde (PFA; Sigma) in PBS for 15 min. at room temperature. After washing with PBS containing 0.05% Tween-20 (PBS-T, BioRad, Hercules, CA), aggregates were treated with ice cold 100% MeOH for 10 min. at -20°C, and washed 3 times with PBS-T before blocking with 0.1% TritonX-100 (Sigma), 1% BSA (Sigma) and 10% Donkey serum (Calbiochem, La Jolla, CA) in PBS for 1 hour at room temperature. The primary antibody was applied and cultured overnight at 4°C. The aggregates were then washed 6 times for 30 min with PBS-T, and incubated with the secondary antibody for 1 hour at room temperature. After being washed 6 times for 30 min with PBS-T at room temperature, the aggregates were then mounted for microscopy with Prolong Gold anti-fade reagent (Invitrogen). Where indicated, the nuclei were stained with DRAQ5 (Cell Signaling, Danvers, MA), diluted (1:1000) in PBS-T, for 20 min. at room temperature before final PBS-T washing. The antibodies used were rabbit anti-Six2 (11562-AP; Proteintech, Chicago, IL), rabbit anti-Laminin (L-9393; Sigma), mouse anti-E-Cadherin (61081; BD Bioscience), rabbit anti-cleaved caspase3 (9664; Cell Signaling Technology, Danvers, MA), mouse anti-pancytokeratin (C2562;Sigma), rabbit anti-Lef1 (2230P; Cell signaling), APC conjugated rat anti-podocalyxin (FAB1556A; R&D systems, Minneapolis, MN), donkey Alexa Fluor 488 anti-rabbit IgG (A21206; Invitrogen), Goat Alexa Fluor 647 anti-rabbit IgG (A21245; Invitrogen), donkey Alexa Fluor 488 anti-mouse IgG (A21202; Invitrogen), donkey Alexa Fluor 647 anti-mouse IgG (A31571; Invitrogen) and donkey Alexa488 Fluor anti-goat IgG (A11055; Invitrogen). For lectin staining, biotinylated DBA (B-1035; Vector Laboratories, Burlingame, CA), biotinylated LTL (B-1325; Vector Laboratories) and TRITC conjugated Neutravidin (A6373; Invitrogen) were used. For EdU staining, Click-iT Plus EdU assay kit (Invitrogen) was used. Experiments requiring the use of two rabbit antibodies were conducted using Zenon dual labeling kits (Invitrogen). Immunostained samples were examined on a laser confocal microscope (FV1000, Olympus).

### Cell sorting and analyses

Cell sorting and analyses were performed by fluorescence activated cell sorter (FACS; Jazz, BD Science). In all experiments where FACS was used to separate different fractions of cells, the purity was always greater than 95% in positive fractions and close to 100% in negative fractions. Dispersed cells were prepared from embryonic kidneys and sorted by the respective GFP or Venus fluorescent signals. Dispersed cells were also prepared from cultured aggregates to analyze Six2-expressing cell populations. For such purpose, dispersed cells were prepared from aggregates by treatment with 0.25% Trypsin/EDTA at 37°C for 5min. After adding DMEM with 10% FBS, the dispersed cells were collected by centrifugation, re-suspended in 2% PFA in PBS, and placed on ice for 20 min. After washing with PBS, cells were again placed on ice in 0.5% saponin (SIGMA, St. Louis, MO) and 1% BSA in PBS for 30–60 min, followed by incubation with Six2 antibody at room temperature for 1 hour. After washing with PBS, cells were again incubated with donkey Alexa488 anti-Rabbit IgG at room temperature for 30 min. and analyzed with Accuri flow cytometer (BD Bioscience). The total number of cells was obtained by counting aliquots of cells by flow cytometer and the number of Six2^+^ cells was obtained by multiplying the percentage of Six2^+^ cells with total number of cells.

### Quantitative PCR

Cultured aggregates were removed from the filter by gentle scraping with forceps, and the total RNA was extracted using Ultraspec RNA isolation system (BIOTECX, Houston, TX). The extracted total RNA was reverse-transcribed using ThermoScript Reverse Transcriptase (Invitrogen) and quantified with PCR thermocycler (MyiQ cycler; BioRad) using iQ SYBR Green Supermix (BioRad). The sequences of primers used are as following (forward/reverse):

Gapdh (TGAACGGATTTGGCCGTATTG/ACCATGTAGTTGAGGTCAATGAAG);

Six2 (CAAGTCAGCAACTGGTTCAAGA/ACTGCCATTGAGCGAGGA);

Cited1 (CCGTACCTCAGCTCCTGTG/AGCTGGGCCTGTTGGTCT);

Eya1 (ATGGAAATGCAGGATCTAAC/AACTTCGGTGCCATTGGGAG);

Gdnf (CGCTGACCAGTGACTCAAT/GCCGCTTGTTTATCTGGTGA)

Wnt4(AGTGGAGAACTGGAGAAGTG/TGTCAAGATGGCCTTCCTG)

Foxd1 (TTCGGATTCTTGGACCAGAC/CAAGTCAGGGTTGCAGCATA);

Slug (GGGGAAAAGCCTTTCTCTTG/TTGGAGCAGTTTTTGCACTG);

Podxl1 (TCCTTGTTGCTGCCCTCTAC/TTCCAAGGTTGGGTTGTCAT);

Slc5a1 (CTTGATCATCTCCTTCCTCACC/ATTGTGTCCTTGGAGTCCTCTG);

Nkcc2 (GATGCAGAACTGGAAGCAGTC/GGCTCTGGAGTGTTCCTGTAAG);

Slc12a3 (ATGATGGCTTCAAGGACGAG/TCCCGAGAGTAATCCAGCAG);

Hes1 (GGCGAAGGGCAAGAATAAAT/GAATGCCGGGAGCTATCTTT);

Hesr1 (GGTACCCAGTGCCTTTGAGA/GTGCGCGTCAAAATAACCTT);

Hesr3 (ATAGAGAAACGGCGCAGAGA/GGCATGGAGCATCTTCAAGT).

### Statistical analysis

All values are expressed as the mean ± S.D. from at least triplicate experiments. Student’s t test for paired and unpaired comparison, as appropriate, was performed and differences were considered to be significant when p < 0.05.

### Ethics Statement

This study was carried out in strict accordance with the recommendations in the Guide for the Care and Use of Laboratory Animals of the National Institutes of Health. The protocol was approved by the IACUC of the VAGLAHS (Permit Number: 01002–09).

## Results

### NPC maintained in re-aggregate system

**Fig 1:**
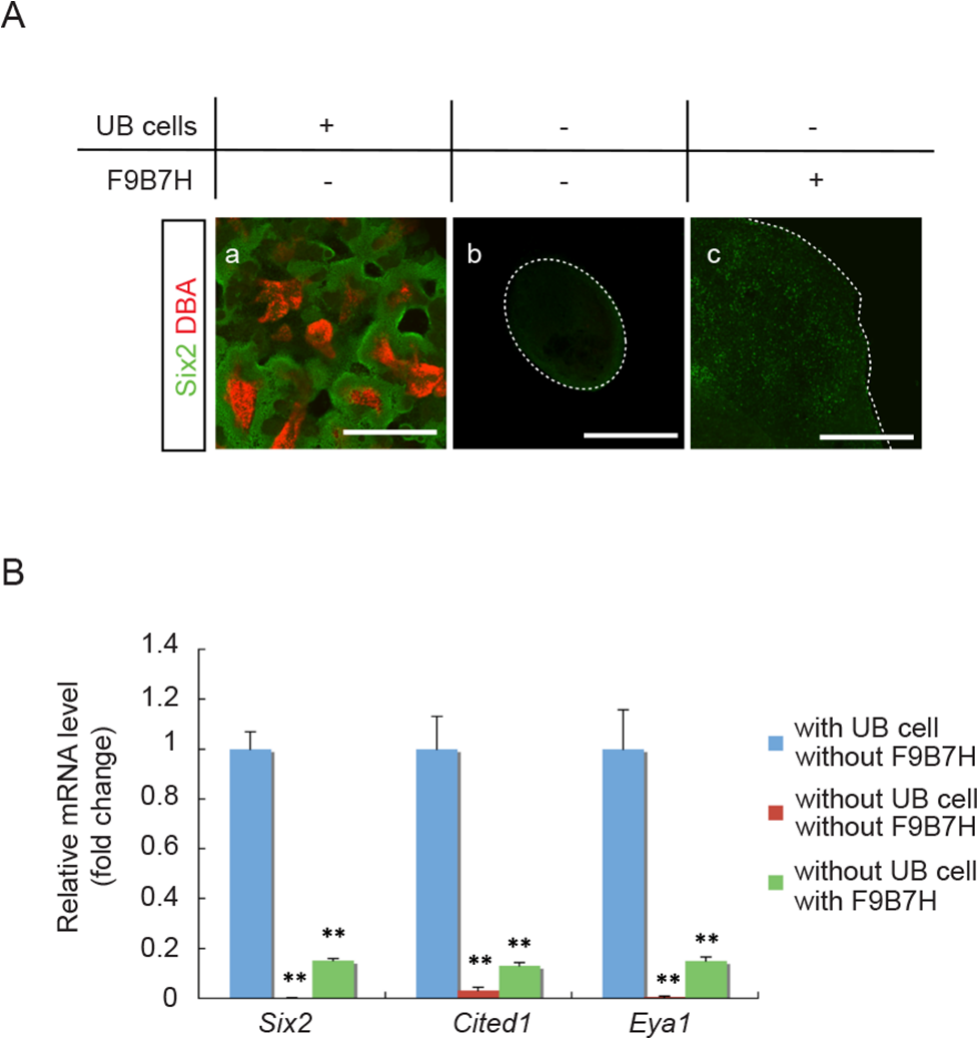
NPC maintenance in aggregates requires UB cells. **(A)** Immuno-staining of E11.5 aggregates for NPC marker, Six2 (green), and UB marker, DBA (red). (a) A representative aggregate made from dispersed E11.5 whole embryonic kidney cells and cultured for 7 days. Abundant Six2^+^ NPC were present surrounding UB structures. (b) A representative aggregate without UB cells reconstituted from E11.5 Hoxb7-Venus mouse embryonic kidneys by manually separating Venus^+^-UB from the surrounding Venus^-^-mesenchyme cells. No Six2^+^ NPC were detected after cultured for 7 days. (c) A representative aggregate without UB cells and treated with Fgf9, Bmp7 and heparin (F9B7H) for 7 days in culture. Only a few Six2^+^ NPC were detected. (Scale bar = 500 μm). **(B)** qRT-PCR results showed significantly lower mRNA expression levels for NPC marker genes (*Six2*, *Cited1* and *Eya1*) in aggregates without UB cells that were either treated or not treated with Fgf9, Bmp7 and heparin (F9B7H). Data were normalized by *Gapdh* expression levels and presented as fold changes from aggregates reconstituted from whole embryonic kidneys that contained UB cells. (n = 3, ** p < 0.01 vs. aggregates with UB cells)

We tested the feasibility of maintaining NPC in *in vitro* culture over an extended period of time using the “re-aggregate” system. As shown in Fig 1Aa, when aggregates were reconstituted with dispersed cells derived from whole E11.5 mouse embryonic kidneys and cultured *in vitro* for 7 days, we were able to detect abundant Six2-expressing (Six2^+^)-NPC surrounding the UB structures. However, when aggregates were reconstituted without UB cells by manually separating Venus^+^-UB from the surrounding Venus^-^-mesenchyme in E11.5 Hoxb7-Venus mouse embryonic kidneys, no Six2^+^-NPC could be detected after 7 days in culture (Fig 1A). Thus, the maintenance of Six2^+^-NPC in the “re-aggregate” system requires the presence of UB cells. Since the combination of Fgf9 and Bmp7 was suggested to be the niche signal capable of maintaining NPC for up to 5 days [17], we tested the effect of adding Fgf9, Bmp7 and heparin to the aggregates without UB cells. As shown in Fig 1Ac, although the addition of these factors was effective to maintain a few Six2^+^-NPC after 7 days in culture as compared to the untreated UB-free samples, the abundance of Six2^+^-NPC was clearly less than that in the untreated aggregates containing UB cells. The mRNA expression levels of NPC markers, including *Six2*, *Cited1* and *Eya1*, were also significantly higher in the aggregates that contained UB cells than those without UB cells, with or without treatment with Fgf9/Bmp7/heparin (Fig 1B).

**Fig 2:**
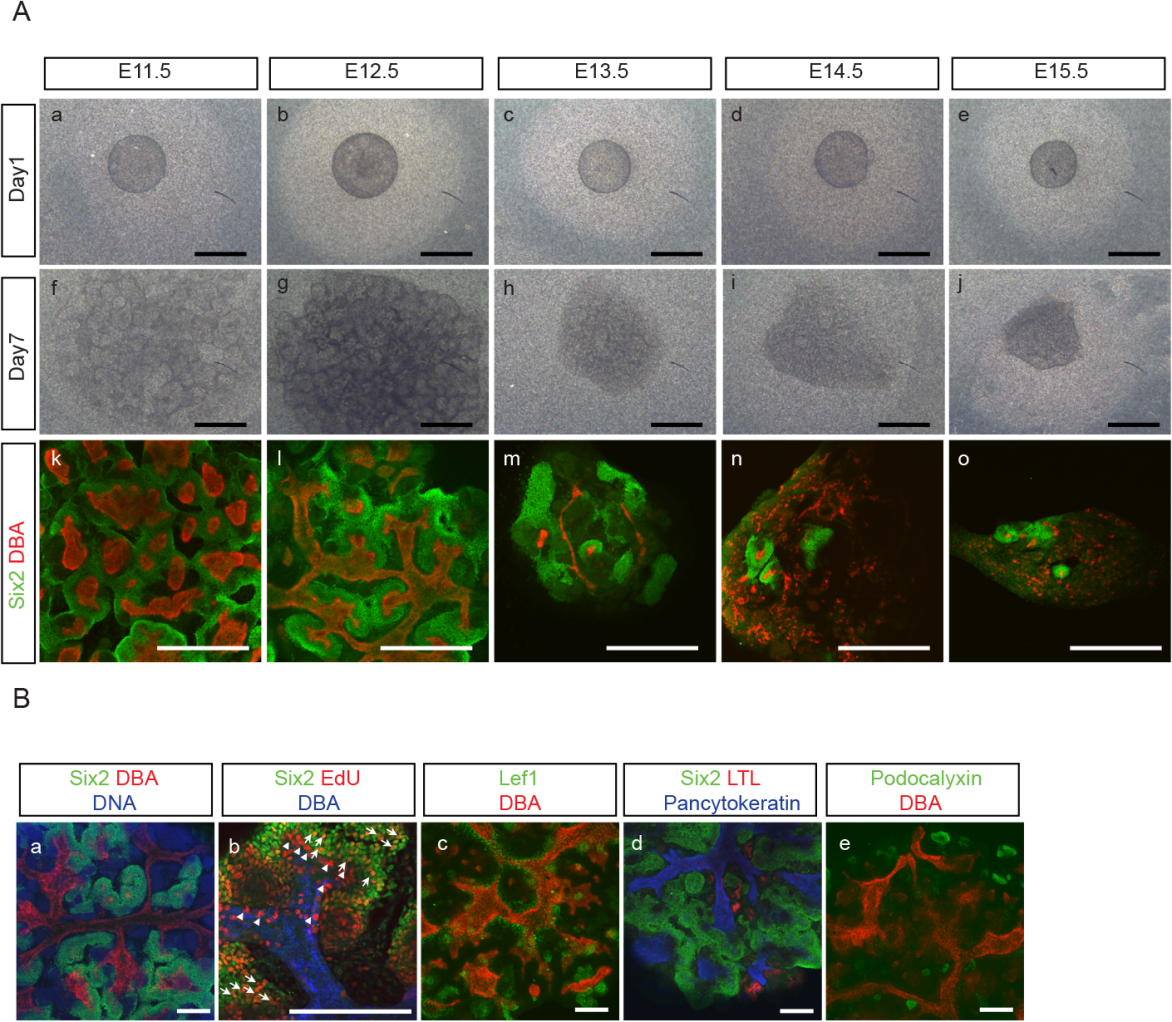
Developmental stage affects NPC maintenance and UB branching morphogenesis in aggregates. **(A**) Aggregates were reconstituted from embryonic kidneys at different developmental stages, starting from E11.5 up to E15.5, and immuno-stained for Six2 (green) and DBA (red) after 7 days in culture. Aggregates from E11.5 (a,f,k) and 12.5 (b,g,l), showed similar characteristics in Six2^+^-NPC maintenance and UB branching morphogenesis. In contrast, aggregates from later developmental stages, i.e., at E13.5 (c,h,m), 14.5 (d,i,n) and 15.5 (e,j,o), showed less well maintenance of Six2^+^-NPC and failure of UB cells to reorganize into orderly branching structures. (Scale bar = 500 μm). **(B)** (a) A representative E12.5 aggregate after 7 days in culture was immuno-stained for NPC marker, Six2 (green), UB marker, DBA (red) and DNA (blue). Abundant Six2^+^ NPC were seen surrounding UB structures, with Six2^-^/DBA^-^ cells in adjacent areas. (b) EdU (5-ethynyl-2’-deoxyuridine) staining of a representative E12.5 aggregate showed Six2^+^/EdU^+^ cells in MM areas that were both close to and distant from the UB structure (white arrow). In contrast, DBA^+^/EdU^+^ cells were detected mostly in UB tips surrounded by Six2^+^-NPC cells (white arrowhead). (c-e) A representative aggregate was immuno-stained for: (c) renal vesicle marker, Lef1 (green)/UB marker, DBA (red); (d) proximal tubule marker, LTL (red)/epithelial marker, pancytokeratin (blue)/NPC marker, Six2 (green); (e) podocyte marker, podocalyxin (green)/UB marker, DBA (red), and showed the development of Lef^+^-renal vesicle like structures (c), LTL^+^- (d) and podocalyxin^+^-(e) epithelial structures. (Scale bar = 200 μm)

We found that aggregates re-constituted from E11.5 and E12.5 embryonic kidneys showed similar characteristics in terms of Six2^+^-NPC maintenance and UB branching morphogenesis (Fig 2A). However, when aggregates were re-constituted from embryonic kidneys at later developmental stages, from E13.5 up to E 15.5, we found that Six2^+^-NPC were not maintained as well and UB failed to reorganize into orderly branching structures (Fig 2A). We therefore used E12.5 embryonic kidneys for the rest of our experiments.

With aggregates reconstituted from E12.5 embryonic kidneys after 7 days in culture, we evaluated cell proliferation pattern by using 5-ethynyl-2’-deoxyuridine (EdU) staining [28, 29]. We detected Six2^+^/EdU^+^ cells in MM areas that were both close to and distant from the UB structure (Fig 2B). In contrast, DBA^+^/EdU^+^ cells were detected mostly in UB tips surrounded by Six2^+^-NPC cells (Fig 2B). We also found the presence of renal vesicle-like structures that expressed Lef1 (Fig 2B), as well as epithelial structures that expressed LTL, a proximal tubular cell marker (Fig 2B), and podocalyxin, a podocyte marker (Fig 2B). These results thus indicate that the re-aggregation system allows not only the proliferation and maintenance, but also the differentiation, of NPC.

In contrast to Six2^+^-NPC, we found that the populations of UB and SM cells decreased over time in the aggregates. For these experiments, we used Hoxb7-Venus [26] and Foxd1-GFP [27] mice for UB and SM cell markers, respectively. As shown in Table 1, while the percentage of Six2^+^-NPC out of the total cells remained unchanged at day 7, Hoxb7-Venus^+^ UB cells decreased by half, and Foxd1-GFP^+^ SM cells disappeared completely after 7 days in culture.

**Table 1:** The percentage of Six2^+^ NP cells, Hoxb7^+^ UB cells and Foxd1^+^ SM cells out of total cells in E12.5 embryonic kidneys at Day0 and in aggregates after 7 days culture without treatment or with treatment with either DMSO or DAPT (n = 3, ** p < 0.01 vs. DMSO treated group).

	Day0	Day7		
		Treatment		
		None	DMSO	DAPT
Six2^+^ NP Cells	34.1 ± 2.0%	34.4 ± 7.6%	28.0 ± 2.7%	56.9 ± 11.8%**
Hoxb7^+^ UB Cells	13.7 ± 3.0%	7.5 ± 0.3%	8.2 ± 0.7%	15.6 ± 0.3%**
Foxd1^+^ SM Cells	20.6 ± 1.9%	0%	0%	0%

**Fig 3:**
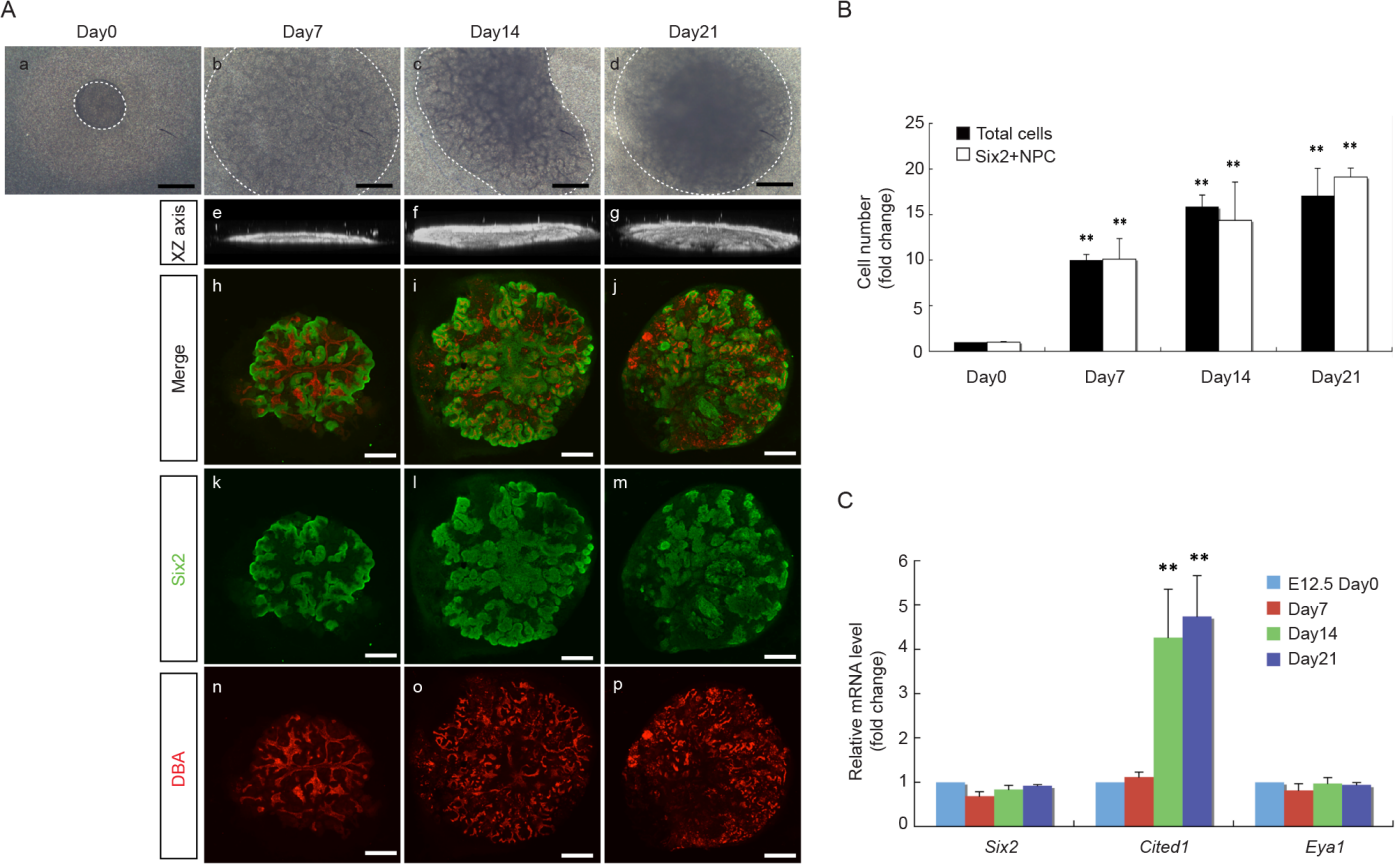
Long term maintenance of NPC in aggregates. **(A)** Morphology of representative E12.5 aggregates at day1, 7, 14 and 21 in culture. The size of aggregates increased with time (a-d), and the morphology of the aggregates changed from more flattened by day 7 to more three-dimensional after day 14, as demonstrated by the confocal XZ axis pictures (e-g). Immuno-staining for NPC marker, Six2 (green), and UB marker, DBA (red), shows the expansion of Six2^+^ NPC surrounding UB structures from day 7 up to day 21 in culture. (Scale bar = 500 μm). **(B)** The number of total cells and Six2^+^NPC in E12.5 aggregates increased in parallel from day 0 to day 7, 14 and 21, and plateaued after day 14. Data are presented as fold changes from respective cell number at day 0. (n = 3, ** p < 0.01 vs. day 0). **(C)** mRNA expression levels of NPC marker genes in E12.5 aggregates at day 0, 7, 14 and 21. qRT-PCR results showed a significant increase in *Cited1* mRNA expression levels at day 14 and 21, while the mRNA expression levels of *Six2* and *Eya1* remained unchanged from day 0 to 21. Data were normalized by *Gapdh* expression levels and presented as fold changes from day 0. (n = 3, ** p < 0.01 vs. day 0)

We further extended the culture period for up to 21 days, a time period when all NPC would have otherwise diminished *in vivo* [30]. We noticed that, while the size of the aggregates increased with time, the morphology of the aggregates changed from more flattened by day 7 to more three-dimensional after day 14 (Fig 3A). As shown in Fig 3A, we detected further expansion of Six2^+^-NPC in the aggregates after 21 days in culture. The extent to which Six2^+^-NPC proliferated over this period of time paralleled to that of the total cells in the aggregates and reached a plateau after day 14 (Fig 3B). The reason for the cell numbers to reach plateau after day 14 appears to be mainly due to a decrease in cell proliferation without changes in cell apoptosis (S1 Fig). qRT-PCR analyses for NPC markers showed a significant increase in *Cited1* mRNA expression levels at days 14 and 21 as compared to the original E12.5 embryonic kidneys at day 0, while the mRNA expression levels of *Six2* and *Eya1* remained unchanged over this period of time (Fig 3C). These results show that the “re-aggregate” system can overcome the *in vivo* limitations and maintain NPC for at least up to 21 days *in vitro*.

### Passages and developmental stage affect the maintenance of NPC

**Fig 4:**
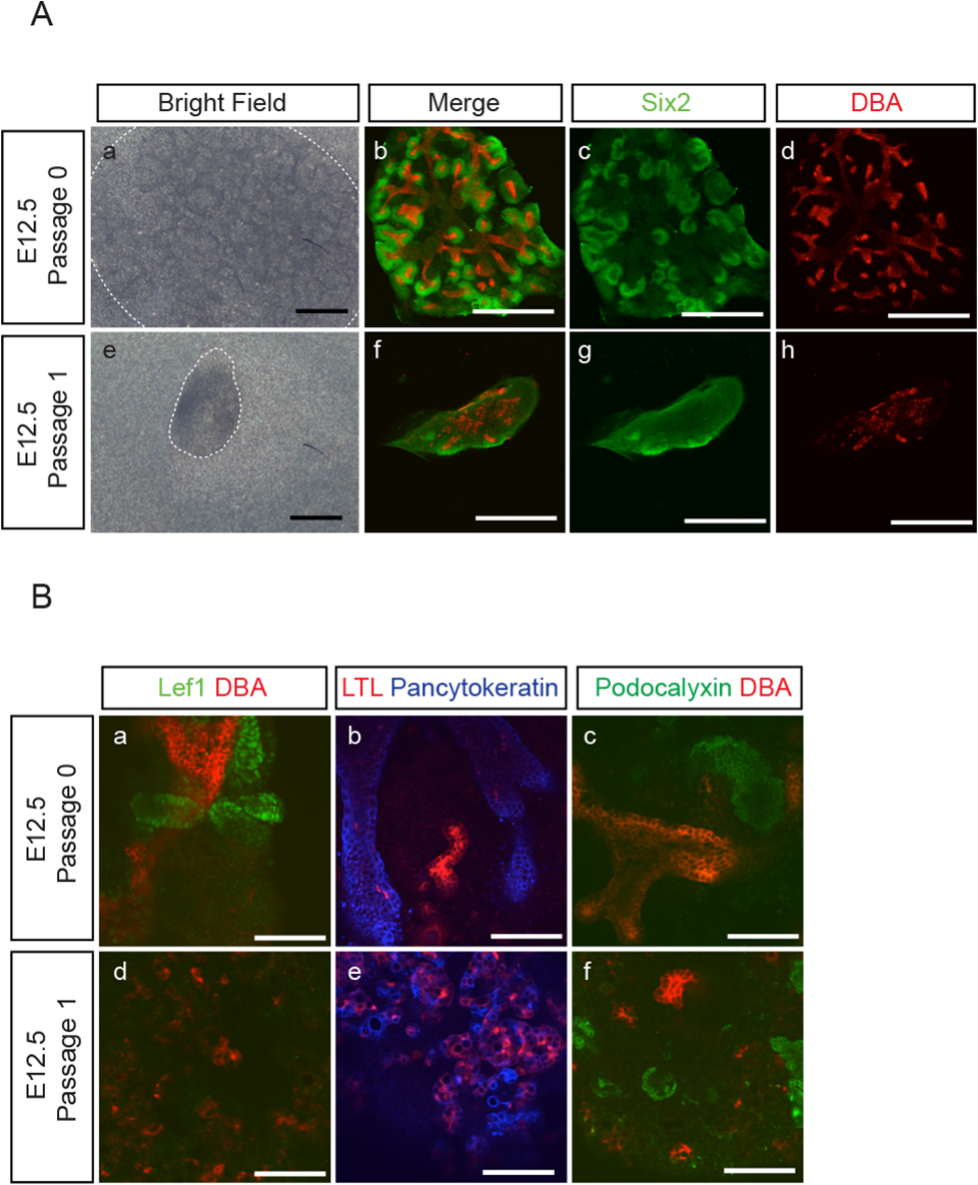
Suboptimal NPC maintenance in P1 aggregates. **(A**) Aggregates were reconstituted from E12.5 embryonic kidneys and cultured for 7 days. Some of the E12.5 P0 aggregates were further passaged at day 7 to reconstitute P1 aggregates and cultured for another 7 days. The bright field images and the immune-staining for NPC marker, Six2 (green), and UB marker, DBA (red), of representative P0 (a-d) and P1 (e-h) aggregates show that P1 aggregates were smaller in size and contained fewer numbers of Six2^+^ NPC as compared to P0 aggregates. In contrast to the organized UB branching structure in P0 aggregates (b, d), UB cells in P1 (f, h) aggregates formed randomly scattered structures. (Scale bar = 500 μm). **(B)** E12.5 P0 and P1 aggregates were immuno-stained for: (a,d) renal vesicle marker, Lef1 (green)/UB marker, DBA (red); (b,e) proximal tubule marker, LTL (red)/epithelial marker, pancytokeratin (blue); (c,f) podocyte marker, podocalyxin (green)/UB marker, DBA (red), and show the development of Lef^+^-renal vesicle like structures (a), LTL^+^- (b,e) and podocalyxin^+^-(c,f) epithelial structures. (Scale bar = 100 μm)

Since the number of Six2^+^-NPC plateaued after 14 days in culture, we tested the possibility to further expand these cells by passage subculture. We first cultured E12.5 aggregates (P0) for 7 days and then dissociated them into single cells to reconstitute new aggregates (P1) at an equal cell number as P0 aggregates. As shown in Fig 4Aa and e, we found that the size of the resultant P1 aggregates after subculture for 7 days was smaller than P0 aggregates at day 7. Although we could still detect some Six2^+^-NPC in P1 aggregates, their abundance in P1 aggregates was markedly less than that in the P0 aggregates (Fig 4A). We also noticed that, unlike the more organized branching structures seen in P0 aggregates (Fig 4A), the UB cells in P1 aggregates formed randomly scattered structures (Fig 4A). The renal vesicle like structures that expressed renal vesicle marker, Lef1, was detected in E12.5 aggregates (P0) at day 7 (Fig 4B), while the epithelial structures that expressed proximal tubule marker, LTL, or podocyte marker, podocalyxin, were detected in both P0 and P1 aggregates at day 7 (Fig 4B).

The reason why P1 aggregates could not maintain Six2^+^-NPC as well as P0 aggregates is not immediately clear. However, we noticed that these P1 aggregates bear close resemblance to aggregates reconstituted from embryonic kidneys at later developmental stage, i.e., E14.5 and 15.5 (Fig 2A), where Six2^+^-NPC were not well maintained and UB cells failed to form organized branching structures. We therefore reasoned that a potential difference between P0 and P1 aggregates could be the more advanced developmental stage of the cell components in P1 aggregates that may interfere with the maintenance of NPC.

### Developmental stage of non-UB populations affects the maintenance of NPC

**Fig 5:**
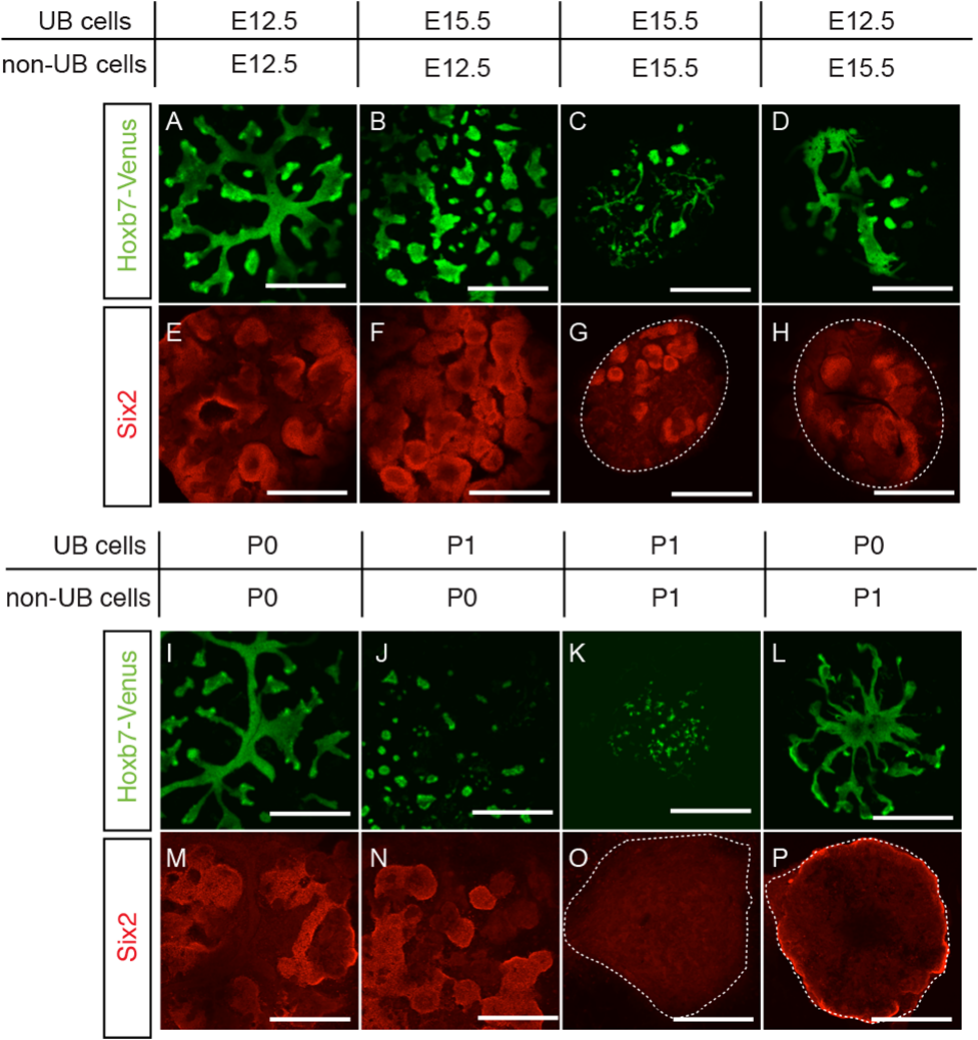
NPC maintenance depends on the developmental stage of non-UB cells. Using Hoxb7-Venus mice, four different combinations of aggregates were reconstituted with respective UB and non-UB cells from E12.5 and E15.5 embryonic kidneys (A-H) or from P0 and P1 E12.5 aggregates (I-P). Representative aggregates after 7 days in culture are shown with Venus^+^-UB structures (green) and immune-stained Six2^+^-NPC (red). Abundant Six2+-NPC were seen in both aggregates with E12.5 (E, F) and P0 (M, N) non-UB cells, but not in aggregates with either E15.5 (G, H) or P1 (O, P) non-UB cells, indicating that NPC maintenance depends on the developmental stage of non-UB cells. On the other hand, more organized UB branching structures were seen in both aggregates with both E12.5 (A, D) and P0 (I, L) UB cells but not in aggregates with either E15.5 (B, C) or P1 (J, K) UB cells, indicating that the formation of organized UB branching structures depends on the developmental stage of UB cells. (Scale bar = 500 μm)

In view of the requirement of UB cells for the maintenance of NPC, and the fact that UB cells in both P1 and E15.5 aggregates failed to form organized branching structures, we tested the possibility that the more developmentally advanced UB cells in E15.5 aggregates could have affected the maintenance of NPC. We first separated UB (Venus^+^) and non-UB (Venus^-^) cells from both E12.5 and E15.5 Hoxb7-Venus embryonic kidneys by fluorescence activated cell sorting (FACS), and then combined UB population (3,000 cells) with non-UB population (20,000 cells) from either E12.5 or E15.5 embryonic kidneys to reconstitute aggregates that resulted in four different combinations as shown in Fig 5. We found that, irrespective of the developmental stage of non-UB populations, all aggregates consisting of E15.5 UB cells developed randomly scattered UB structures (Fig 5B and 5C), while aggregates consisting of E12.5 UB cells developed more organized branching structures (Fig 5A and 5D). On the other hand, we also found that, irrespective of the developmental stage of UB cells, and therefore irrespective of UB branching structures, abundant Six2^+^-NPC were maintained only in those aggregates consisted of E12.5 non-UB cells (Fig 5E and 5F).

In parallel to these results from E15.5 embryonic kidneys, we found that combinations of UB and non-UB cells from either P0 or P1 aggregates at day 0 gave similar results, i.e., Six2^+^-NPC were maintained only with P0 non-UB cells independent of the passage of UB cells (Fig 5M and 5N), while the formation of more organized branching UB structures were observed with P0 UB cells independent of the passage of non-UB cells (Fig 5I and 5L). These results indicate that the formation of organized UB branching structures is dependent on the developmental stage of UB cells, while the maintenance of Six2^+^-NPC is dependent on the developmental stage of the non-UB cell populations.

### Differentiated MM cells interfere with NPC maintenance

**Fig 6:**
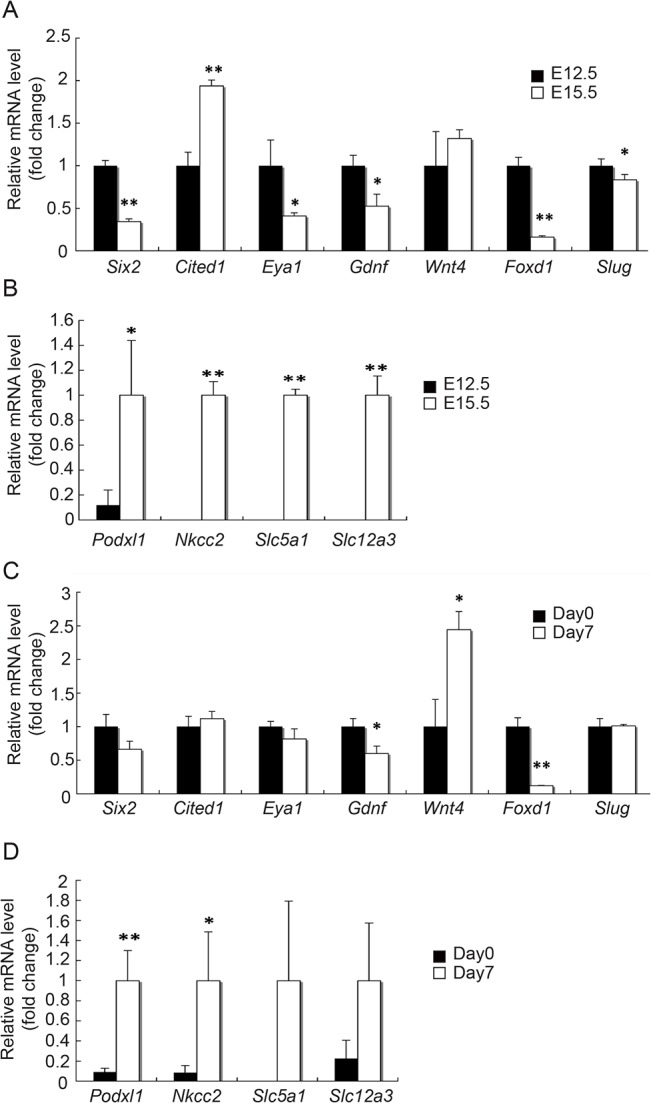
Expression of differentiated MM markers in E15.5 embryonic kidneys and E12.5 aggregates after 7 days in culture. qRT-PCR results show that, as compared to E12.5 embryonic kidneys, E15.5 embryonic kidneys had a significant decrease in the expression of NPC marker genes (*Six2*, *Eya1* and *Gdnf*) and a significant increase in the expression of differentiated MM cell marker genes (*Podxl1*, *Nkcc2*, *Slc5a1* and *Slc12a3*) (A, B). A similar increase in the expression of these differentiated MM cell marker genes was also found with E12.5 aggregates after 7 days in culture (C, D). There was also a significant decrease in the expression of SM cell marker gene (*Foxd1*) in both E15.5 embryonic kidneys and E12.5 aggregates after 7 days in culture. Data were normalized by *Gapdh* expression levels and presented as fold changes from:(A) E12.5 embryonic kidneys, (B) E15.5 embryonic kidneys, (C) E12.5 aggregates at day 0, (D) E12.5 aggregatesafter 7 days in culture. (n = 3, * p<0.05, ** p < 0.01)

To further explore the reason why Six2^+^-NPC were not maintained in aggregates containing E15.5 non-UB cells, we analyzed and compared the expression profiles of non-UB cell marker genes between E12.5 and E15.5 embryonic kidneys. As shown in Fig 6A, we found that E15.5 embryonic kidneys showed significantly lower expression levels of NPC markers, such as *Six2* and *Eya1*, as compared to E12.5 embryonic kidneys, although the expression of another NPC marker *Cited1* was significantly elevated (Fig 6A). The expression of differentiated MM cell markers, such as *Podxl1*, *Nkcc2*, *Slc5a1* and *Slc12a3*, were also significantly increased in E15.5 non-UB cells (Fig 6B). Nevertheless, the expression of SM cell markers, such as *Foxd1* and *Slug*, was significantly decreased in E15.5 non-UB cells (Fig 6A). Similarly, we found a significant increase in the differentiated MM cell markers, including *Poxdl1*, *Nkcc2*, *Slc5a1* and *Slc12a3*, and a significant decrease in SM cell marker *Foxd1*, in the E12.5 aggregates after *in vitro* culture for 7 days, as compared to E12.5 embryonic kidneys at day 0 (Fig 6C and 6D). The decrease in *Foxd1* expression level is consistent with the disappearance of Foxd1-GFP^+^ cells in E12.5 aggregates after 7 days in culture (Table 1). These results raised the possibility that the inability to maintain NPC in aggregates containing either E15.5 or P1 non-UB cells could be due to the decrease of SM cells or the presence of differentiated MM cells.

**Fig 7:**
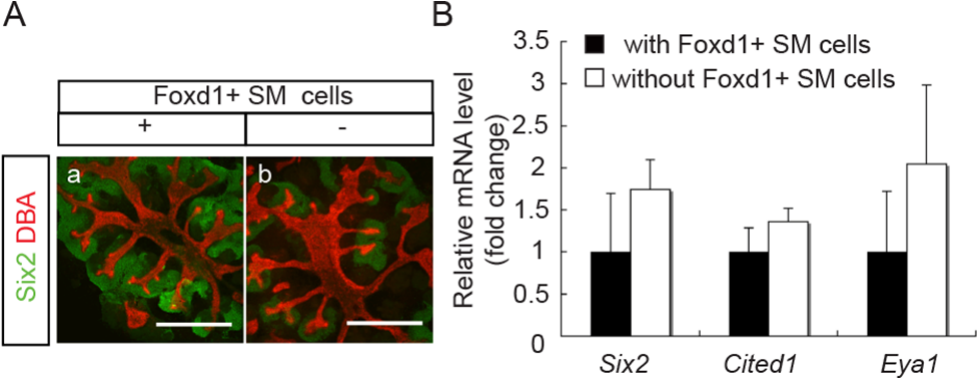
No difference between E12.5 aggregates with or without Foxd1^+^ SM cells. Aggregates without Foxd1^+^ SM cells were produced from Foxd1-EGFP mouse E12.5 embryonic kidneys after Foxd1^+^ SM cells were removed by FACS sorting, and compared to similar aggregates containing SM cells after 7 days in culture. **(A)** Immuno-staining of representative aggregates for NPC marker, Six2 (green), and UB marker, DBA (red), shows no difference in the abundance of Six2^+^NPC and UB structures between the two (Scale bar = 500 μm). **(B)**qRT-PCR results also show no difference in the mRNA expression levels of NPC marker genes (*Six2*, *Cited1* and *Eya1*). Data were normalized by *Gapdh* expression levels and presented as fold changes from aggregates containing Foxd1^+^ SM cells.

In view of the recently proposed role of SM cells as promoting MM cell differentiation [6,7], it seems unlikely that the decrease in SM cells in E15.5 or P1 aggregates could be accountable for the inability to maintain NPC in these aggregates. Nevertheless, we tested this possibility by making aggregates without Foxd1^+^ SM cells with FACS sorted GFP^-^ populations from E12.5 Foxd1-GFP mouse embryonic kidneys. As shown in Fig 7A, we found no difference in the abundance of Six2^+^-NPC between aggregates with or without Foxd1^+^ SM cells after 7 days in culture. The expression of NPC markers, *Six2*, *Cited1* and *Eya1*, also was not different between the two groups (Fig 7B). These results negate the possibility that a reduced Foxd1^+^ SM cell population in E15.5 or P1 aggregates could have caused their inability to maintain Six2^+^-NPC.

**Fig 8:**
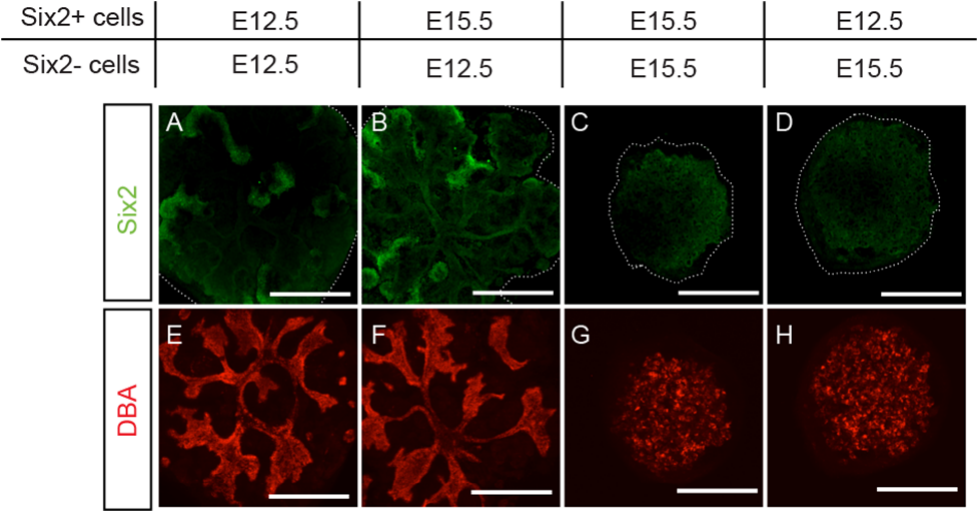
NPC maintenance depends on the developmental stage of non-NPC cells. Using Six2-GCE mice, four different combinations of aggregates were reconstituted with respective Six2^+^ and Six2^-^ cells from E12.5 and E15.5 embryonic kidneys (A-H). Representative aggregates after 7 days in culture were immuno-stained for Six2 (green) and DBA (red). More abundant Six2^+^-NPC were seen in both aggregates reconstituted with E12.5 Six2^-^ cells (A, B), indicating that Six2^+^-NPC maintenance depends on the developmental stage of Sxi2^-^-non-NPC. Organized UB branching structures were seen only in aggregates reconstituted with E12.5 Six2^-^ cells (E, F). Note that nuclear Six2 signals were detected only in aggregates reconstituted with E12.5 Six2^-^ cells (A, B) (Scale bar = 500 μm)

Since we found in E15.5 embryonic kidneys a significantly lower expression level of NPC markers and a significantly higher expression level of differentiation markers as compared to E12.5 embryonic kidneys (Fig 6A and 6B), we first tested the possibility that the presence of differentiated MM cells in E15.5 aggregates could have affected the maintenance of NPC. For this purpose, we used Six2-GCE mice (see S2 Fig) to separate Six2-GFP^+^ and Six2-GFP^-^ cells from both E12.5 and E15.5 embryonic kidneys by FACS, and then combined the Six2-GFP^+^ populations (8000 cells) with Six2-GFP^-^ populations (20,000 cells) to reconstitute aggregates that resulted in four different combinations as shown in Fig 8. We found that, irrespective of the developmental stage of Six2-GFP^+^ populations, all aggregates consisted of E15.5 Six2-GFP^-^ cells developed randomly scattered UB structures (Fig 8G and 8H), while those aggregates consisted of E12.5 Six2-GFP^-^ cells developed more organized branching structures (Fig 8E and 8F). On the other hand, we found that, irrespective of the developmental stage of Six2-GFP^+^ cells, more abundant Six2^+^-NPC were maintained in those aggregates consisted of E12.5 Six2-GFP^-^ cells than aggregates consisted of E15.5 Six2-GFP^-^ cells (Fig 8A and 8B).

**Fig 9:**
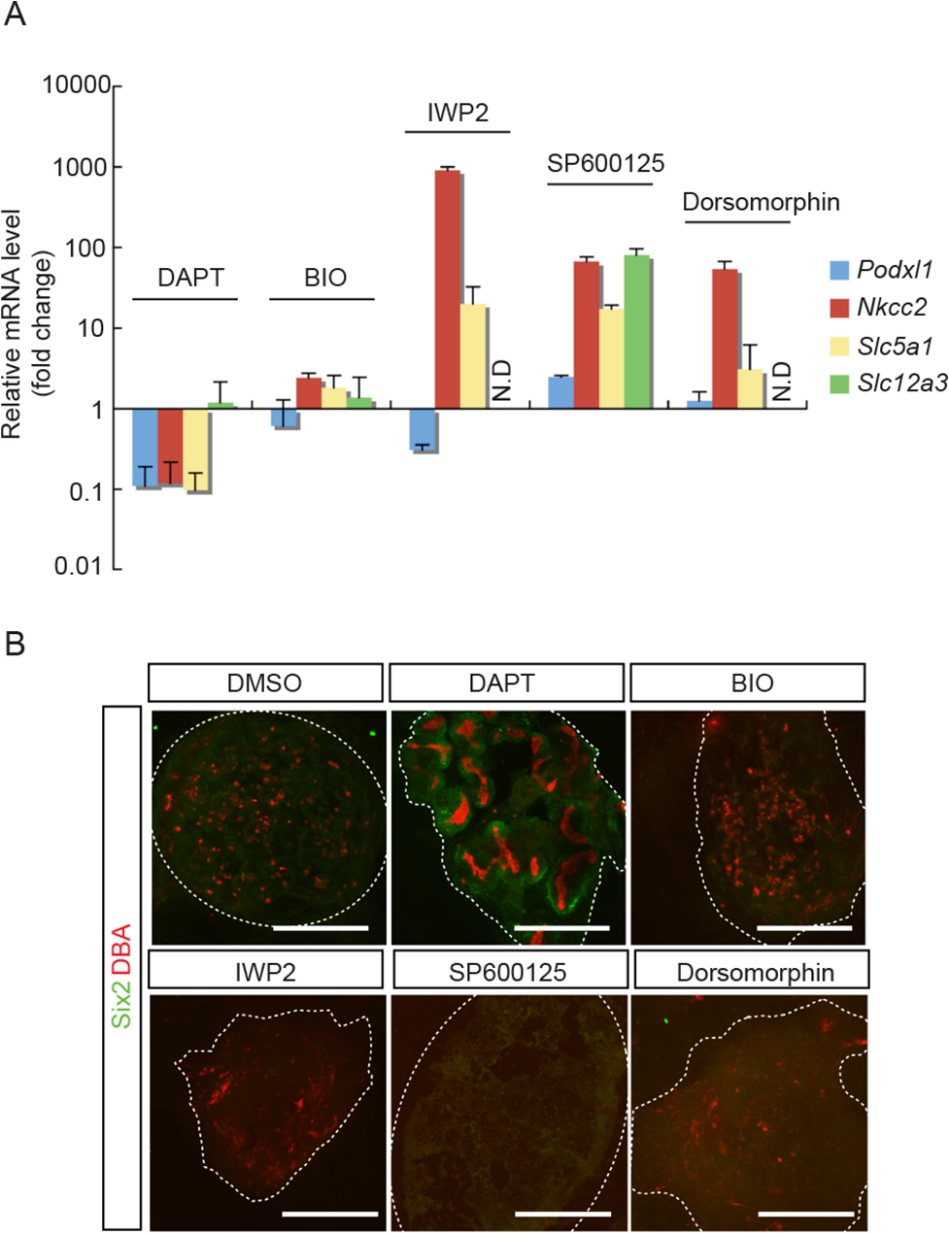
DAPT prevented MM cell differentiation in E12.5 embryonic kidneys during organ culture and maintained NPC in aggregates derived therefrom. **(A)** E12.5 embryonic kidneys were treated with various inhibitors for 7 days in organ culture. qRT-PCR results show the effect of Notch signal inhibitor (DAPT) in suppressing the expression of MM differentiation marker genes (*Podxl1*, *Nkcc2* and *Slc5a1*, but not *Slc12a3*). No effects were seen with Wnt signal inhibitor (IWP2), GSK3β inhibitor (BIO), JNK inhibitor (SP600125) or BMP signal inhibitor (Dorsomorphin). Data were normalized by *Gapdh* expression levels and presented as fold changes from DMSO treated embryonic kidneys as control. (N.D: not detected). **(B)** E12.5 embryonic kidneys were treated with different inhibitors for 7 days in organ culture. Cells were then dispersed from these embryonic kidneys to reconstitute aggregates and cultured for another 7 days without respective inhibitors. Immuno-staining of representative aggregates for NPC marker, Six2 (green), and UB marker, DBA (red), shows the presence of Six2^+^-NPC only in aggregates derived from DAPT-pretreated embryonic kidneys. (Scale bars = 500 μm)

Having ruled out UB and SM cell components in our above mentioned experiments, these results led us to suspect that the differentiated MM cells, which were contained in the E15.5 Six2-GFP^-^ fraction, may interfere with NPC maintenance. To verify this possibility, we tested the effects of inhibitors on different signaling pathways known to be involved in MM differentiation. The inhibitors we tested include: IWP2, a Wnt signaling pathway inhibitor [31]; BIO, a GSK3β inhibitor [32]; SP600125, a JNK inhibitor [33]; Dorsormorphin, a Bmp signaling pathway inhibitor [34]; and *N*-*S*-phenyl-glycine-*t*-butyl ester (DAPT), a γ-secretase inhibitor that inhibits the Notch signaling pathway [35]. We first verified the effects of these inhibitors on the expression of differentiated MM cell markers in E12.5 embryonic kidneys under conventional organ culture for 7 days. As shown in Fig 9A, we found that, as compared to DMSO treated control samples, only DAPT was effective in reducing most of the differentiated MM cell markers, such as *Podxl1* for podocytes and *Nkcc2*, *Slc5a1* for proximal tubular cells. The distal tubular cell marker, *Slc12a3*, remained unchanged. These results reflect the known role of the Notch signaling pathway in directing MM cell differentiation towards podocyte and proximal tubules [35]. In parallel, we reconstituted aggregates with cells dispersed from these organ-cultured embryonic kidneys and examined the maintenance of Six2^+^-NPC in these aggregates after another 7 days of culture in the absence of respective inhibitors. As shown in Fig 9B, we were able to detect Six2^+^-NPC only in the aggregates prepared from DAPT-treated embryonic kidneys. Taken together, these results suggest that the inability of E15.5 aggregates to maintain NPC may in part be due to the direct and/or indirect interference from differentiated MM cells.

### DAPT treatment enhances NPC maintenance

**Fig 10:**
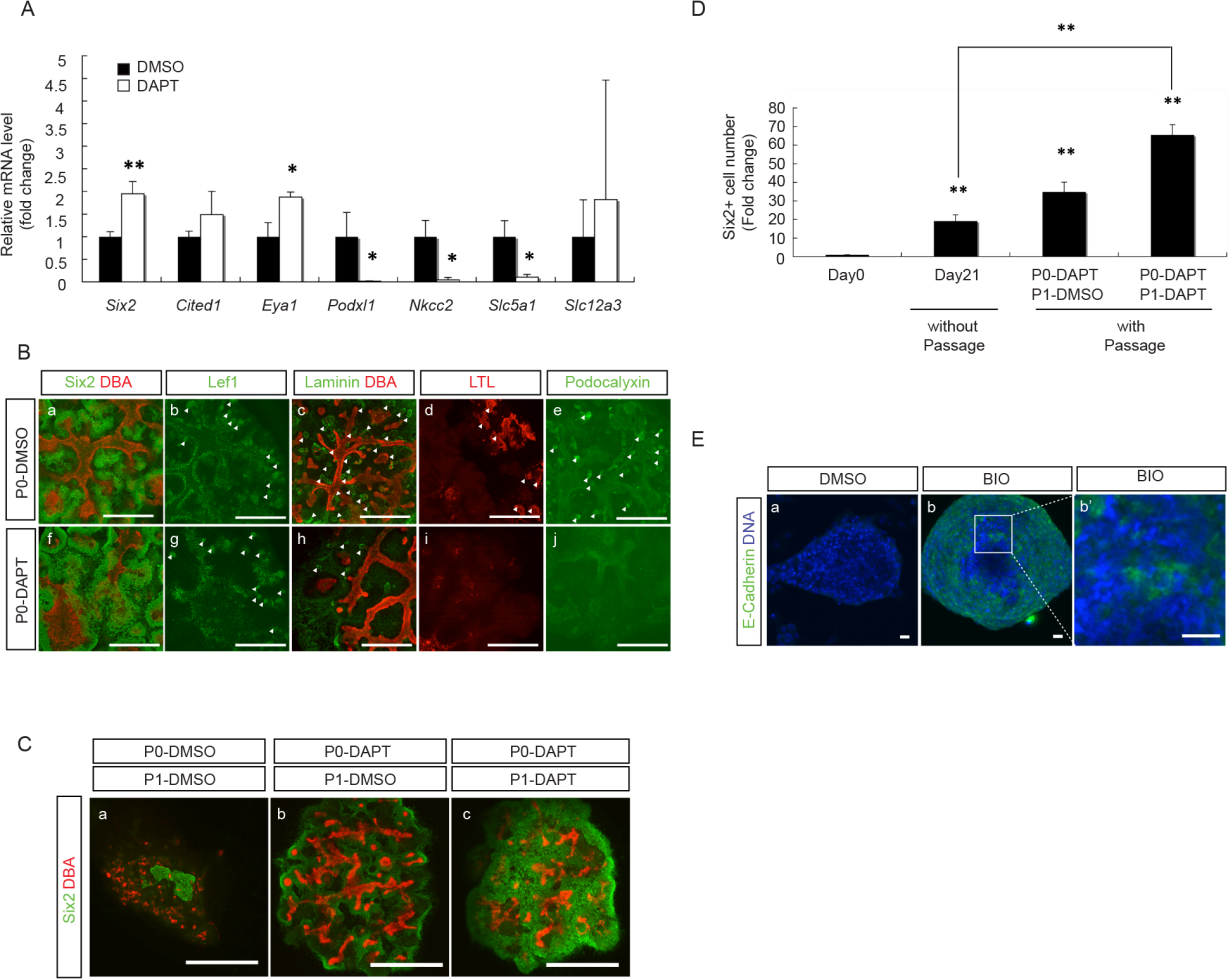
DAPT treatment enabled further expansion of NPC in P1 aggregates. **(A)** qRT-PCR results from E12.5 aggregates after treatment with either DMSO or DAPT for 7 days show a significant increase in NPC marker genes (*Six2* and *Eya1*) and a significant decrease in differentiated MM cell marker genes (*Podxl1*, *Nkcc2* and *Slc5a1*, but not *Slc12a3*) in DAPT-treated aggregates as compared to DMSO-treated aggregates. All data were normalized by *Gapdh* expression levels and presented as fold changes from DMSO treated. (n = 3, * p < 0.05, ** p < 0.01). **(B)** Immuno-staining of representative E12.5 aggregates, after 7 days in culture with either DMSO or DAPT, for: NPC marker, Six2 (green)/UB marker, DBA (red) (a,f); renal vesicle marker, Lef1 (green) (b,g); epithelial marker laminin (green)/UB marker, DBA (red) (c,h); proximal tubule marker, LTL (red) (d,i); podocyte marker, pdocalyxin (green) (e,j), shows increased abundance of Six2^+^ NPC(f), with reduced laminin^+^- (h), LTL^+^- (i) and podocalyxin^+^- (j) epithelial structures in DAPT-treated aggregates as compared to DMSO-treated aggregates. White arrowheads indicate structures that are positive for respective markers. (Scale bar = 500μm). **(C)** Immuno-staining of representative P1 aggregates that were treated with either DMSO or DAPT during P0 and P1 periods for NPC marker, Six2 (green), and UB marker, DBA (red), shows that DAPT-treatment during P0 period alone increased the abundance of NPC in P1 aggregates (b vs. a). The number of NPC was further expanded by continuing DAPT treatment during both P0 and P1 periods (c vs. a&b). (Scale bar = 500μm). **(D)** Six2^+^-NPC number increased by 20-fold in aggregates without passage after 21 days in culture as compared to that in the E12.5 embryonic kidneys at day 0. This was further increased to 35- and 65-fold by passage with DAPT treatment during P0 period alone and during both P0 and P1 periods, respectively. (n = 3, ** p < 0.01 vs. day 0 and day 21). **(E)** Aggregates from Six2-TGC mouse E12.5 embryonic kidneys were treated with DAPT during both P0 and P1 periods, and the Six2-GFP^+^ cells from these P1 aggregates were collected by FACS sorting and used to reconstitute aggregates for another 24 hour incubation with either DMSO or BIO. After being cultured for another 5 days without treatment, these aggregates were immuno-stained for epithelial cell marker, E-cadherin (green). Results from two representative aggregates show that treatment with BIO (b, b’), but not DMSO (a), induced E-cadherin expression, indicating that Six2^+^ NPC from these P1 aggregates retained their potential to respond to Wnt signal to become epithelial cells. (b’) is a higher power view of (b) showing the induced E-cadherin^+^-epithelial structures by BIO treatment. (Scale bar = 20 μm)

Extrapolating the above findings from E12.5 embryonic kidneys, we tested the effect of DAPT treatment in P0 aggregates. As shown in Fig 10A, treatment of E12.5 aggregates with DAPT for 7 days up-regulated NPC markers, including *Six2* and *Eya1*, and down-regulated podocyte and proximal tubule markers, such as *Podxl1* and *Nkcc2*, *Slc5a1*, respectively. The distal tubule marker, *Slc12a3*, remained unchanged. Similar to E12.5 embryonic kidney organ culture results, we found abundant Six2^+^-NPC in DAPT-treated P0 aggregates, which was associated with a decrease in the epithelial structures with the expression of Laminin, a nephron epithelial cell marker, as well as LTL, a proximal tubular cell marker and podocalyxin, a podocyte marker, while the Lef^+^-renal vesicle like structures remained less affected (Fig 10B). As shown in Table 1, the percentage of Six2^+^-NPC out of the total cell population increased by 2-fold as compared to the DMSO-treated control group. The proportion of Hoxb7^+^ UB cells also increased by 2-fold, and the Foxd1^+^ SM cells remained undetectable.

Based on these results, we further tested the possibility of DAPT treatment to maintain NPC in P1 aggregates. We first cultured E12.5 P0 aggregates with or without DAPT for 7 days, and then dissociated cells from these aggregates to reconstitute P1 aggregates and continued culture for another 7 days with or without DAPT. As shown in Fig 10C, DAPT treatment during P0 culture alone maintained more Six2^+^-NPC in the subsequently reconstituted P1 aggregates after being cultured for another 7 days with DMSO (Fig 10C), than the P1 aggregates derived from DMSO-treated P0 aggregates and continued culture for 7 days with DMSO (Fig 10C). When we continued DAPT-treatment with P1 aggregates derived from DAPT-treated P0 aggregates, we detected an even greater expansion of Six2^+^-NPC (Fig 10C). As shown in Fig 10D, the continued DAPT treatment throughout P0 and P1 periods allowed the continued expansion of Six2^+^-NPC after passage, resulting in an increase in the number of Six2^+^-NPC up to 65 times greater than that in the original E12.5 embryonic kidneys at day 0. This also represents an increase by 3-fold as compared to the E12.5 aggregates at day 21 without passage.

To confirm that the Six2^+^-NPC maintained in P1 aggregates preserved their potential to respond to the induction by Wnt signal, we tested the effect of GSK3β inhibitor, BIO, on aggregates reconstituted with Six2^+^-NPC from P1 aggregates. For these studies, we used E12.5 aggregates with dispersed cells from Six2-TGC mouse (see S2 Fig) embryonic kidneys. After DAPT treatment during both P0 and P1 periods, the Six2-EGFP^+^ cells in the final P1 aggregates were FACS sorted to reconstitute new aggregates for further culture with either DMSO or BIO for another 7 days. As shown in Fig 10E, BIO treatment induced epithelial structures with the expression of E-cadherin, an epithelial cell marker, in these reconstituted aggregates. These results support the preserved potential of Six2^+^-NPC in DAPT-treated P1 aggregates to respond to Wnt signal and transform to epithelial cells.

## Discussion

In our present study, we demonstrated that the “re-aggregate” system can be used to maintain and expand NPC in *in vitro* culture over prolonged period of time. We found that aggregate formation is essential, because NPC could not be maintained when we simply mixed the dispersed cells from whole E12.5 embryonic kidneys and cultured them in 2D culture plates (data not shown). We also found that the maintenance of NPC in this system required the presence of UB cells (Fig 1A). It is therefore likely that this "re-aggregate" system provides a 3D platform that enables UB cells to form niche structures and release niche signals to keep NPC within proximity and promote their proliferation. Recent studies have unraveled many candidates of the UB-derived niche signals, which include Fgfs (2 and 9) [16, 17], BMP7 [18] and Wnt9b [19]. Fgfs were shown to promote the survival of NPC through Fgf receptors, Fgfr 1 and 2 [16, 17], and BMP7 was shown to promote the proliferation and maintaining the self-renewal of NPC [18]. Indeed, the combination of Fgf9 and Bmp7 has been proposed to be the UB niche signals capable of maintaining NPC for up to 5 days in *in vitro* culture [17]. However, in our studies, we found that the combination of Fgf9 and Bmp7 alone was not as effective as UB cells (Fig 1A). This could conceivably be due to the fact that these factors, when added to the culture medium, may not permeate far enough to reach the inner part of the aggregates. It is also possible that there are additional UB cell-derived niche signals that work together with Fgf9 and Bmp7 to maintain NPC at the niche. In this regard, Wnt9b expressed in the developing UB was proposed to be capable of regulating the balance between differentiation and maintenance of NPC through its effect to induce different target genes in NPC in a Six2-dependent manner [19]. Further studies will be required to evaluate the role of Wnt9b/β-catenin signaling in the maintenance of NPC in “re-aggregate” system.

With this "re-aggregate" system, we found that we were able to maintain NPC for at least up to 21 days. The number of Six2^+^-NPC gradually increased to reach almost 20-fold by 21 days, but the increase appeared to have plateaued after 14 days (Fig 3C). We found that this was mainly due to a decrease in cell proliferation without changes in cell apoptosis (S1 Fig). Nevertheless, we found that the majority of Six2^+^ cells at day 21 were also positive for EdU. This would indicate that the Six2^+^ NPC remained at day21 were capable of self-renewal with continuing proliferation while maintaining their Six2^+^ status. In an attempt to sustain the proliferation of NPC before it reached to a plateau, we tried passage subculture by making dispersed cells from the original P0 aggregates at day 7 and reconstituting them into new P1 aggregates. However, we found that these P1 aggregates did not grow well and did not maintain Six2^+^-NPC as well as P0 aggregates (Fig 4). These features with P1 aggregates were also found in aggregates made from E15.5 embryonic kidney cells (Fig 2), leading us to suspect that the more advanced developmental stage of cell populations in P1 aggregates may be accountable for their differences from P0 aggregates. The expression of differentiated MM marker genes in both E15.5 embryonic kidneys and P0 aggregates at day 7 (Fig 6) further supported this notion.

To delineate which of the three cell components in the aggregates, i.e., UB cells, SM cells and MM cells, could have contributed to the changes found in E15.5 and P1 aggregates, we reconstituted different combinations of aggregates using respective UB and non-UB populations from either E12.5 and E15.5 embryonic kidneys or P0 and P1 aggregates (Fig 5). Results from these experiments showed that the maintenance of Six2^+^-NPC depended mainly on the developmental stage of the non-UB cells; while the ability of the UB cells to form organized branching structures depended mainly on the developmental stage of UB cells. Although the reason why UB cells in both E15.5 and P1 aggregates could not form organized branching structures awaits further investigation, the fact that these UB cells were able to maintain Six2^+^-NPC would indicate that the scattered UB structures in E15.5 and P1 aggregates were sufficient to function as UB niches.

Another feature that we found common in both E15.5 embryonic kidneys and P0 aggregates after 7 days in culture was a dramatic decrease in Foxd1^+^ SM cells (Table 1 and Fig 6). Although we did not observe an increase in Slug expression (Fig 6), we cannot rule out the possibility that the decrease in Foxd1^+^ cells could be due to their further differentiation along the SM cell lineage and lost the expression of Foxd1. Nevertheless, the decrease in Foxd1^+^ SM cells seems unlikely to be the cause of the inability of E15.5 and P1 aggregates to maintain NPC, because Foxd1^+^ SM cells have recently been found to rather promote NPC differentiation [6,7], and indeed Foxd1 null mutants are known to be associated with an expansion of cap mesenchymal cells [7,36]. Consistent with this notion, our results show no significant difference between aggregates with and without Foxd1^+^ SM cells, although we did not detect an obvious expansion of Six2^+^-NPC in our aggregates without Foxd1^+^ SM cells after 7 days in culture.

In support of the possibility that the differentiated MM cells in E15.5 and P1 aggregates may interfere with NPC maintenance, we found that we were able to sustain Six2^+^-NPC proliferation in P1 aggregates by inhibiting the Notch signaling pathway. The Notch family, *Notch1-4*, are transmembrane receptors that mediate short-range communication between cells [37]. Upon binding of ligands, *Jag1 and Dll1*, expressed on adjacent cells, the intracellular domain of *Notch* protein is released through γ-secretase-mediated proteolysis and translocate to the nucleus to regulate target gene transcription [38]. Notch signaling molecules, particularly *Notch 1* and *Notch 2*, are expressed throughout kidney development [38] and *Notch* 2 is known to play an important role in nephron formation, including the differentiation of MM cells towards proximal tubule and podocyte [39]. It is also interesting to note that, besides its role in determining cell fate, the Notch signaling pathway may affect NPC self-renewal through the suppression of Six2 expression [40]. Consistent with these reports, we found that DAPT treatment prevented differentiation of NPC and allowed their continuing expansion with passage in P1 aggregates. This enabled a harvest of 65 times more Six2^+^-NPC than the starting material, and an additional three times more than aggregates without passage. These Six2^+^-NPC from DAPT treated P1 aggregates maintained their potential to respond to the induction by the Wnt signal. While we have confirmed the effect of DAPT to inhibit Notch signaling pathway by the down-regulation of Notch down-stream Hairy genes, *Hes1*, *Hesr1* and *Hesr3* (S3 Fig), we cannot exclude the possibility that the Notch-independent γ-secretase inhibition effect of DAPT may have also contributed to the effect of DAPT.

## Conclusions

We describe in our current study an *in vitro* culture system which allows maintenance and expansion of NPC by using the "re-aggregation" system in combination with UB cells and DAPT treatment. This "re-aggregation" system may prove useful not only as a tool for studies on kidney development, but also as an autologous source of kidney replacement therapies.

## Supporting Information

S1 Fig Cell apoptosis and proliferation assays in E12.5 aggregates after long term culture. (A-C, A’-C’) A representative E12.5 aggregate each at days 7, 14 and 21 was immuno-stained for apoptosis marker, cleaved caspase 3 (green), NPC marker, Six2 (blue) and UB marker, DBA (red). No significant difference in apoptosis activity was detected. (D-F, D’-F’) A representative E12.5 aggregate each at days 7, 14 and 21 was immuno-stained for proliferation marker, EdU (red), NPC marker, Six2 (green) and UB marker, DBA (blue). EDU^+^ cells decreased from day 7 to day 14 and 21. EDU^+^ cells were detected in both Six2^+^ and Six2^-^ cells at day 7, but mainly in Six2^+^ cells at days 14 and 21, indicating the continuing self-renewal of Six2^+^ NPC at day 21. (Scale bar = 100μm)
(TIF)

S2 Fig Reduced Six2^+^ NPC in aggregates reconstituted from E12.5 embryonic kidneys from Six2-GCE and Six2-TGC mice. Two different strains of Six2-GFP mouse were used in our present study. One is the Six2-GCE mouse, where EGFP-CreERT2 allele was knocked into the Six2 gene locus, so that GFP^+^ mice are heterozygous with only half of the endogenous Six2 expressed. The other is the Six2-TGC mouse, where EGFP-Cre allele was inserted into Six2 promoter region with BAC transgene and is expected to have no influence on endogenous Six2 expression. Although both strains have normal phenotype *in vivo*, we found that aggregates reconstituted from these two strains of mouse showed abnormal growth and did not maintain Six2^+^ NPC well in culture. (A) After 7 days in culture, the percentage of Six2-GFP^+^ cells decreased significantly in both strains. (n = 3). (B) After 7 days in culture, the total number of cells in E12.5 aggregates from Six2-GCE mice was slightly lower than that from the wild type littermate mice. In contrast, the total number of cells in E12.5 aggregates from Six2-TGC mice was significantly lower than that from the wild type littermate mice. (n = 3, ** p<0.01 vs. wild type). (C) Representative aggregates from Six2-GCE and Six2-TGC mice after 7 days in culture were immune-stained for NPC marker, Six2 (green), and UB marker, DBA (red), show a significantly lower abundance of Six2^+^ cells in both Six2-GCE (b) and Six2-TGC (d) aggregates as compared to the respective wild type (a, c) aggregates. (Scale bar = 500 μm). The reason for the abnormality with the Six2-GCE aggregates could be explained by the reduced expression level of Six2 protein in heterozygous Six2-GCE aggregates and allowed NPC differentiation. The reason for the same abnormality in aggregates from Six2-TGC mice is not immediately clear. One possibility could be the random insertion of EGFP-Cre allele that interfered with functions of not only Six2^+^ cells but also Six2^-^ cells.
(TIF)

S3 Fig DAPT treatment inhibited the expression of Notch downstream genes. qRT-PCR results show that DAPT treatment inhibited the expression of typical downstream genes of Notch signaling, *Hes1* and *Hesr1*, as compared to control treatment with DMSO. (n = 3, ** p < 0.01)
(TIF)
