# Supplementary figures and images for: Maintenance of Mouse Nephron Progenitor Cells in Aggregates with Gamma-Secretase Inhibitor
Source: PLoS One. 2015 Jun 15;10(6):e0129242. doi: 10.1371/journal.pone.0129242 (PMC4468097; doi:10.1371/journal.pone.0129242)

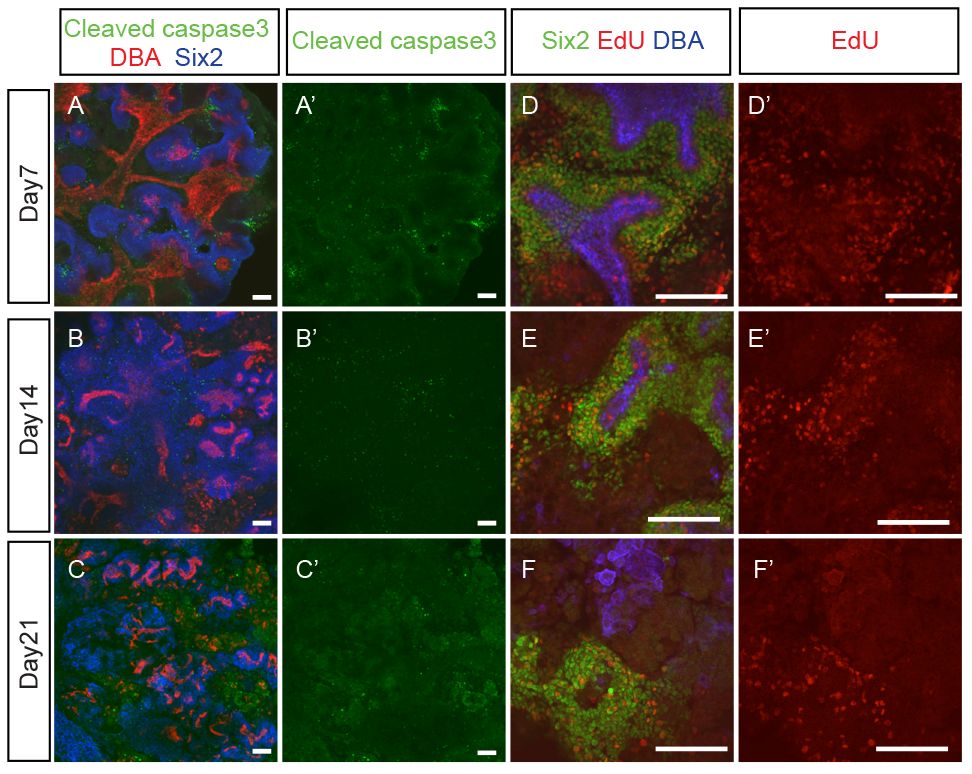

Supplement: S1 Fig — (A-C, A’-C’) A representative E12.5 aggregate each at days 7, 14 and 21 was immuno-stained for apoptosis marker, cleaved caspase 3 (green), NPC marker, Six2 (blue) and UB marker, DBA (red). No significant difference in apoptosis activity was detected. (D-F, D’-F’) A representative E12.5 aggregate each at days 7, 14 and 21 was immuno-stained for proliferation marker, EdU (red), NPC marker, Six2 (green) and UB marker, DBA (blue). EDU+ cells decreased from day 7 to day 14 and 21. EDU+ cells were detected in both Six2+ and Six2- cells at day 7, but mainly in Six2+ cells at days 14 and 21, indicating the continuing self-renewal of Six2+ NPC at day 21. (Scale bar = 100μm) (TIF) [file pone.0129242.s001.tif]

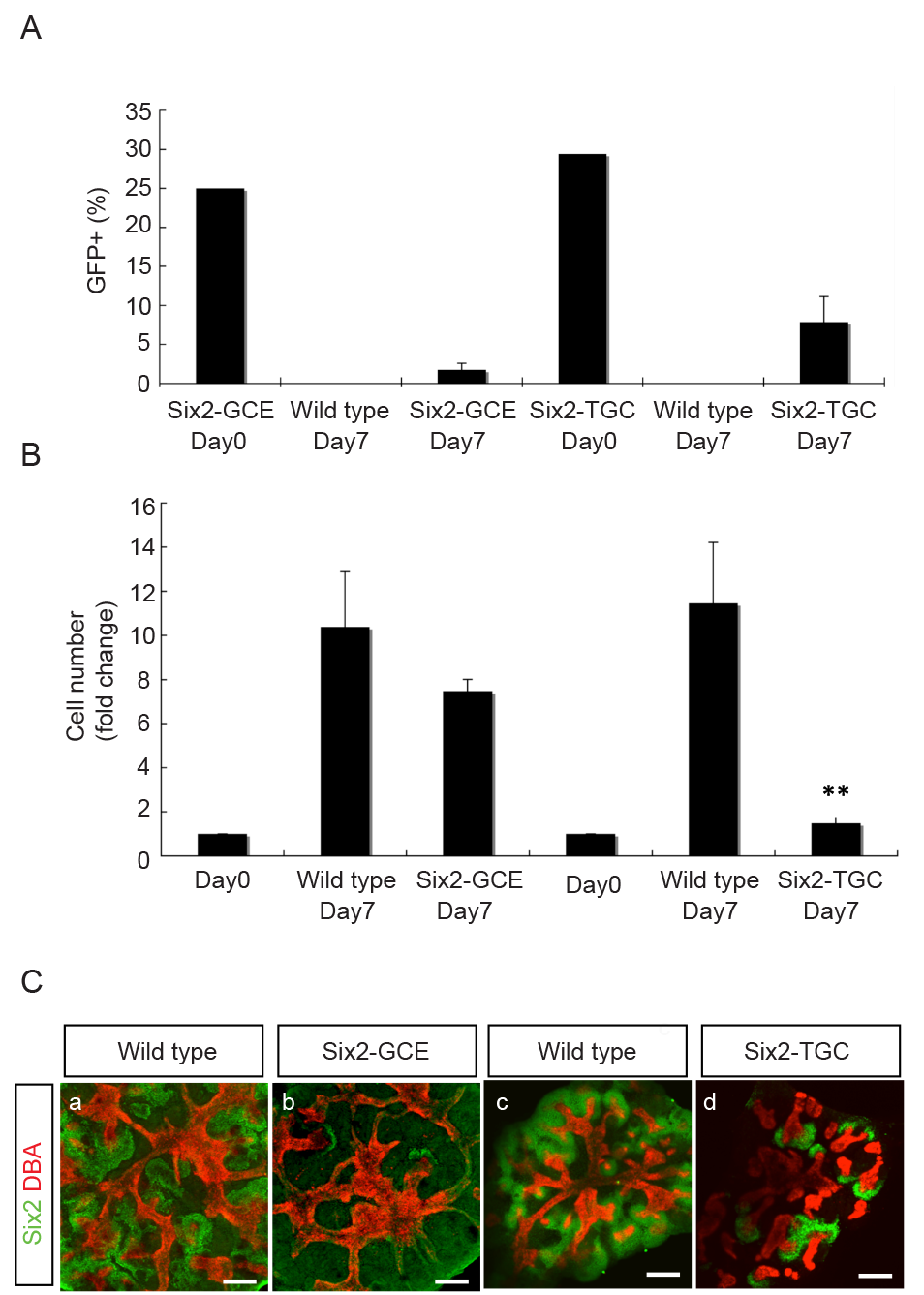

Supplement: S2 Fig — Two different strains of Six2-GFP mouse were used in our present study. One is the Six2-GCE mouse, where EGFP-CreERT2 allele was knocked into the Six2 gene locus, so that GFP+ mice are heterozygous with only half of the endogenous Six2 expressed. The other is the Six2-TGC mouse, where EGFP-Cre allele was inserted into Six2 promoter region with BAC transgene and is expected to have no influence on endogenous Six2 expression. Although both strains have normal phenotype in vivo, we found that aggregates reconstituted from these two strains of mouse showed abnormal growth and did not maintain Six2+ NPC well in culture. (A) After 7 days in culture, the percentage of Six2-GFP+ cells decreased significantly in both strains. (n = 3). (B) After 7 days in culture, the total number of cells in E12.5 aggregates from Six2-GCE mice was slightly lower than that from the wild type littermate mice. In contrast, the total number of cells in E12.5 aggregates from Six2-TGC mice was significantly lower than that from the wild type littermate mice. (n = 3, ** p<0.01 vs. wild type). (C) Representative aggregates from Six2-GCE and Six2-TGC mice after 7 days in culture were immune-stained for NPC marker, Six2 (green), and UB marker, DBA (red), show a significantly lower abundance of Six2+ cells in both Six2-GCE (b) and Six2-TGC (d) aggregates as compared to the respective wild type (a, c) aggregates. (Scale bar = 500 μm). The reason for the abnormality with the Six2-GCE aggregates could be explained by the reduced expression level of Six2 protein in heterozygous Six2-GCE aggregates and allowed NPC differentiation. The reason for the same abnormality in aggregates from Six2-TGC mice is not immediately clear. One possibility could be the random insertion of EGFP-Cre allele that interfered with functions of not only Six2+ cells but also Six2- cells. (TIF) [file pone.0129242.s002.tif]

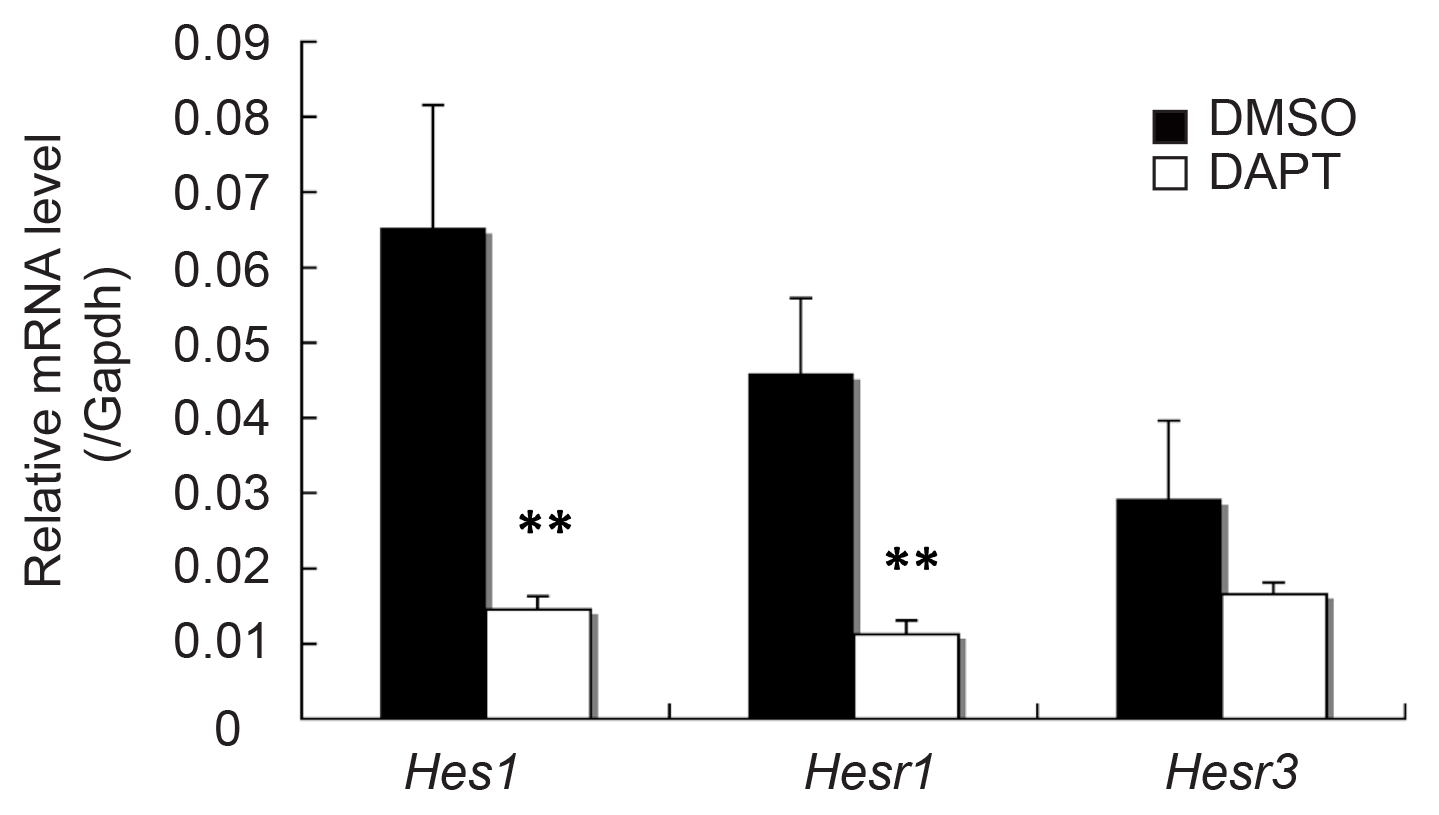

Supplement: S3 Fig — qRT-PCR results show that DAPT treatment inhibited the expression of typical downstream genes of Notch signaling, Hes1 and Hesr1, as compared to control treatment with DMSO. (n = 3, ** p < 0.01) (TIF) [file pone.0129242.s003.tif]
